# A window into the brain: multimodal MRI assessment of vascular cognitive impairment

**DOI:** 10.3389/fnins.2025.1526897

**Published:** 2025-04-16

**Authors:** Changjun Ma, Ailian Liu, Jiahui Liu, Xiulin Wang, Fengyu Cong, Ying Li, Jing Liu

**Affiliations:** ^1^School of Biomedical Engineering, Faculty of Medicine, Dalian University of Technology, Dalian, China; ^2^Stem Cell Clinical Research Center, National Joint Engineering Laboratory, Regenerative Medicine, The First Affiliated Hospital of Dalian Medical University, Dalian, China; ^3^Dalian Innovation Institute of Stem Cell and Precision Medicine, Dalian, China; ^4^Department of Radiology, The First Affiliated Hospital of Dalian Medical University, Dalian, China; ^5^Faculty of Information Technology, University of Jyväskylä, Jyväskylä, Finland

**Keywords:** vascular cognitive impairment, glymphatic system, diffusion tensor imaging, resting-state functional MRI, dynamic functional connectivity, structure–function coupling

## Abstract

Vascular cognitive impairment (VCI) encompasses a diverse range of syndromes, including mild cognitive impairment and vascular dementia (VaD), primarily attributed to cerebrovascular lesions and vascular risk factors. Its prevalence ranks second only to Alzheimer’s disease (AD) in neuro diseases. The advancement of medical imaging technology, particularly magnetic resonance imaging (MRI), has enabled the early detection of structural, functional, metabolic, and cerebral connectivity alterations in individuals with VCI. This paper examines the utility of multimodal MRI in evaluating structural changes in the cerebral cortex, integrity of white matter fiber tracts, alterations in the blood–brain barrier (BBB) and glymphatic system (GS) activity, alteration of neurovascular coupling function, assessment of brain connectivity, and assessment of metabolic changes in patients with VCI.

## Introduction

Vascular cognitive impairment (VCI) primarily arises from vascular risk factors and cerebrovascular diseases (van der Flier et al., 2018; Corriveau et al., 2016), encompassing mild cognitive impairment, vascular dementia (VaD), and varying levels of cognitive decline resulting from mixed pathologies such as comorbid Alzheimer’s disease (AD) (Zhang et al., 2019). VCI is further classified into four subtypes based on stroke history and clinicopathologic/imaging features: post-stroke vascular cognitive impairment (PSCI), subcortical ischemic vascular cognitive impairment (SVCI), multiple infarct vascular cognitive impairment, and mixed dementia with other neurodegenerative pathologies (Iadecola et al., 2019).

**Figure 1:**
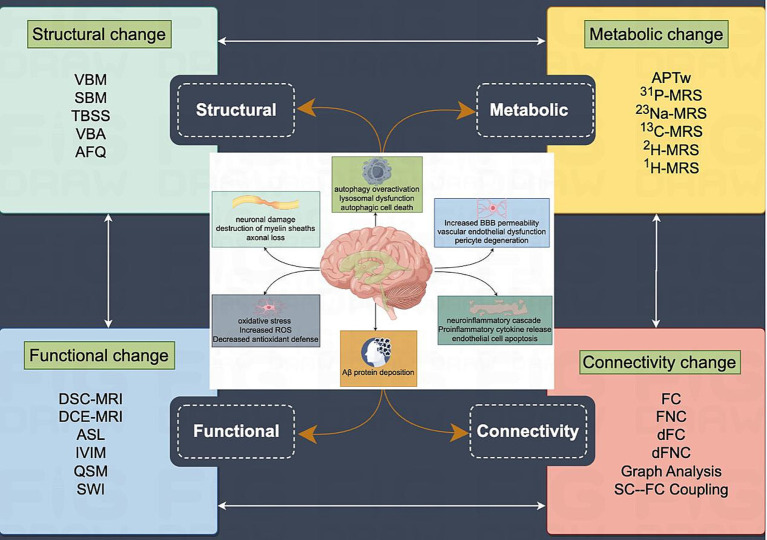
Pathophysiologic alterations and multimodal MRI assessment system for VCI: individuals diagnosed with VCI may exhibit pathophysiological alterations, including neuronal injury, myelin destruction, axonal loss, dysfunction of the neurovascular unit, oxidative stress-induced damage to neuronal cells, and activation of a neuroinflammatory cascade resulting in the formation of a pro-inflammatory microenvironment within the brain. These changes ultimately contribute to modifications in brain structure, function, metabolism, and connectivity. The advancement of MRI imaging technology has enabled the utilization of multimodal MRI and associated post-processing techniques to effectively delineate alterations in brain structure, function, metabolism, and connectivity. Images were produced using FigDraw software (www.figdraw.com) (DOI: PPARW86662).

VCI may arise from various etiologies related to cerebrovascular diseases, such as cerebral small-vessel disease (CSVD), large-artery atherosclerosis, cerebral hemorrhage, cardiogenic embolism, and uncommon stroke etiologies (Dichgans and Leys, 2017). Currently, the etiology of VCI remains uncertain, with potential pathogenic mechanisms including neuronal injury resulting from cerebral hypoperfusion and hypoxia, destruction of myelin sheaths and loss of axons (Rosenberg et al., 2016; Du et al., 2019; Bálint et al., 2019), intracerebral homeostatic imbalance caused by dysfunction of the neurovascular unit such as disruption of the blood–brain barrier (BBB) (Lee et al., 2017; Parfenov et al., 2019), oxidative stress-induced damage to neurons, and the development of an intracerebral pro-inflammatory microenvironment in response to neuroinflammatory cascades (Parfenov et al., 2019; Liu et al., 2018). Moreover, patients with VCI exhibit abnormalities in lipid metabolism (Liu et al., 2020), glucose metabolism (Ma et al., 2018), and amino acid metabolism (Hosoki et al., 2023). Consequently, analyzing alterations in brain microstructure, function, metabolism and connectivity in VCI patients can inform targeted clinical interventions. Nevertheless, the evaluation of cognitive function changes in VCI patients in clinical settings predominantly relies on subjective cognitive scales, which are highly subjective and cannot reflect the changes in brain structure, function, metabolism, and connectivity in the process of cognitive dysfunction in VCI patients. The advancement of imaging technology has led to the utilization of multimodal magnetic resonance imaging (multi-MRI) technology in evaluating cognitive status in patients with VCI. Structural MRI (sMRI) sequences offer insights into brain morphology and structural alterations in VCI patients, while functional MRI (fMRI) can quantitatively analyze changes in cerebral perfusion, BBB permeability, cerebral hypoxic microenvironment, iron deposition, brain function connections, and glymphatic system (GS) activity, etc. in VCI patients. Metabolic MRI can provide accurate and quantitative assessments of glucose, lipid, and amino acid metabolism, as well as detect changes in tissue osmolality, neurotransmitters, mitochondrial function, etc. The integration of multimodal MRI for intelligent analysis of brain connectivity offers a novel approach to evaluating the brain network of patients with VCI in both resting and dynamic states. This review summarizes the current research progress in the utilization of multi-MRI techniques for the diagnosis and treatment of VCI (as shown in Figure 1).

## Evaluation of the integrity of white matter fiber bundles in VCI patients

Chronic hypoperfusion and disruption of the neurovascular microenvironment have been shown to have detrimental consequences on neuronal homeostasis, resulting in neuronal death and chronic demyelination (Du et al., 2019; Liesz, 2019). The deficiency of myelin significantly compromises the integrity of white matter fiber tracts, ultimately leading to impaired cognitive function in the brain (Iadecola, 2013). Therefore, evaluating the integrity of white matter fiber bundles in patients with VCI can provide valuable insights into the degree of cognitive impairment present, thereby informing clinical interventions.

The rapid development of non-invasive neuroimaging technology has spawned a series of novel studies exploring the developmental patterns of the brain. MRI signals are used to obtain information such as image data, index parameters and other information, which can then discriminate the microstructural properties of different tissues. Currently, imaging studies probing tissue microstructure generally favor diffusion tensor imaging (DTI) metrics that respond to the diffusion properties of water molecules in white matter. DTI is a non-invasive neuroimaging technique that allows for the quantification of water molecule diffusion tensors and detailed evaluation of white matter fiber bundles. In recent years, DTI has been extensively utilized for the assessment of white matter microstructure (Le Bihan et al., 2001; Pierpaoli and Basser, 1996). Methods for analyzing the integrity of white matter fiber bundles using DTI primarily encompass region of interest (ROI)-based analysis, fiber bundle-based analysis, and voxel-based analysis. Fiber-based Tracer Spatial Statistical (TBSS) analysis is a voxel-based diffusion tensor imaging (DTI) data analysis technique employed to derive metrics such as fiber length, fiber integrity, and quantitative parameters, including fractional anisotropy (FA) and mean diffusivity (MD), of white matter fiber bundles through the mapping of brain white matter fibers (Smith et al., 2007). In contrast, the voxel-based analysis (VBA) analysis allows for statistical analysis of tensor metrics, including FA and MD values, computed during data preprocessing, to compare between-group differences at the whole-brain voxel level (McWilliam et al., 2023). The TBSS analysis method addresses issues related to alignment errors and smoothing kernel selection that are present in the VBA method, while also mitigating the bias in individual measurements and improving the accuracy of lesion localization compared to hand-drawn ROIs. However, the method’s skeletonization of fiber bundles disrupts the correspondence between diffusion parameters and actual anatomical structures, leading to challenges in result interpretation. Additionally, TBSS is limited in its ability to analyze specific fiber bundles. Furthermore, the TBSS method may not ensure precise analysis due to the potential discontinuity of the white matter skeleton in regions where fibers intersect (Smith et al., 2007). To address the aforementioned challenges associated with fiber bundle analysis in intersection regions, the implementation of automatic fiber quantification (AFQ) offers substantial improvements in the examination of white matter fiber intersections. This is achieved through the processes of fiber bundle segmentation, intersection identification, and the separation and quantification of fiber bundles (Yeatman et al., 2012). Concurrently, fiber bundle quantification, also known as tractometry, effectively captures the local microstructural heterogeneity within intersection regions, thereby facilitating the resolution of multifiber intersections with enhanced robustness and precision (Rheault et al., 2020). Recent research studies (Kim et al., 2016; Chen et al., 2018; Mascalchi et al., 2019; Liu et al., 2019b) have demonstrated that the TBSS analysis method offers a more objective quantitative assessment of white matter fibers, presenting a novel functional imaging evaluation approach for diagnosing and predicting the effectiveness of VCI. Kim et al. (2011) identified a relationship between cognitive and motor impairments and quantitative DTI metrics in individuals with SVCI utilizing the VBA technique. Specifically, they found that cognitive performance was linked to atypical DTI metrics in supratentorial regions, while motor deficits were associated with both supratentorial and infratentorial lesions. Additionally, Zhou et al. (2011) reported that patients with VCI exhibited decreased FA values across the entire brain, indicating more severe white matter damage that correlated with cognitive dysfunction. This was demonstrated through histogram analysis of DTI data and VBA techniques. Another study (Chenet al., 2020) demonstrated that diffusion indices of specific fiber bundles in patients with SVCI were correlated with changes in cognitive function, as determined through AFQ analysis. The DTI-based analysis demonstrates the ability to quantitatively, comprehensively, noninvasively, and objectively assess the integrity of white matter fiber bundles, providing valuable guidance for clinical treatment. However, DTI also has its limitations, its diffusion tensor model cannot effectively measure the white matter properties of fibre crossing regions, while in fact most brain regions contain complex cross-fibre structures. To solve this problem, scholars have proposed methods to improve image acquisition strategies and higher-order models, such as diffusion kurtosis imaging (DKI) and neuronal orientation dispersion and density imaging (NODDI).

DKI sequence offers a more comprehensive assessment of the non-Gaussian diffusion behavior of water molecules compared to DTI sequences, enabling the quantitative evaluation of deviations between actual non-Gaussian diffusion displacements and ideal Gaussian diffusion displacements through kurtosis values. These values serve as indicators of the extent of restriction and heterogeneity in water molecule diffusion (Jensen and Helpern, 2010; Jensen et al., 2005). Liu et al. (2020) observed decreased kurtosis values in the left hippocampus of patient with SVCI, which were inversely associated with cognitive performance scores. Additionally, Fan et al. (2022) observed a decrease in kurtosis values within ROIs across various brain regions in patient with PSCI. These reduced kurtosis values were found to be significantly associated with cognitive scores in the patient population. DKI, with increased sensitivity to intracellular restricted diffusion, enhances the ability to detect subtle microstructural changes in tissues. This heightened sensitivity offers a more reliable method for assessing the integrity of white matter fiber bundles in patients with VCI.

In addition, the NODDI sequence has the advantages of higher specificity, better resolution and multi-parameter assessment compared with the traditional DTI sequence, which can analyse more complex brain tissue microstructures (Zhang et al., 2012). Beck et al. (2021) evaluated the age-dependence of diffusion metrics in the adult life cycle by six different diffusion models, including DTI, DKI and NODDI, and compared the age prediction accuracies of the six different diffusion models, and found that the more advanced diffusion models (e.g., the DKI and NODDI models) were more sensitive to age-related microstructural changes in the brain white matter. Complementing and extending the contribution of traditional DTI models.

## Evaluation of blood–brain barrier permeability and changes in glymphatic system function in individuals with vascular cognitive impairment

The blood–brain barrier (BBB) plays a crucial role in controlling the passage of molecules from the bloodstream to the central nervous system, thereby maintaining the appropriate supply of essential nutrients and oxygen, shielding the brain from potentially harmful neurotoxic compounds circulating in the blood, and enhancing the removal of waste products across endothelial cells (Abbott et al., 2010); dysfunction in the PVS results in inadequate fluid flow within the ISF, impairing the elimination of neurotoxins and promoting the accumulation of detrimental substances (Tarasoff-Conway et al., 2015). In recent academic discourse, scholars have introduced the concept of the GS, a comprehensive brain perivascular fluid transport mechanism responsible for eliminating waste products from the ISF by enabling ISF-cerebrospinal fluid (CSF) exchange through aquaporin-4 (AQP-4) water channels located in the vascular end feet of astrocytes (Iliff et al., 2012; Iliff et al., 2013). Consequently, the functioning of the GS is essential for preserving brain fluid equilibrium across the lifespan. Prior research has demonstrated that reduced CBF and oxygen levels in individuals with VCI result in an increase in BBB permeability, the infiltration of plasma proteins into the perivascular spaces, impaired neurovascular coupling, and dysfunctional GS activity (Lee et al., 2017; Parfenov et al., 2019). Numerous studies have indicated that compromised GS function is correlated with various neurological conditions, such as AD (Rasmussen et al., 2018), diabetes (Jiang et al., 2017), traumatic brain injury (Iliff et al., 2014) and stroke (Gaberel et al., 2014), etc. GS dysfunction has been linked to sleep disorders (Xie et al., 2013) as well as to tau (Harrison et al., 2020) and β-amyloid (Aβ) (Eide et al., 2023) deposition, which are pathogenic mechanisms for cognitive impairment and dementia. In addition, the identification of the GS system has opened up avenues for exploring novel therapeutic approaches and targets, as well as for monitoring drug concentrations in central nervous system disorders where the BBB poses a challenge to effective treatment. Multi-modal MRI offers a noninvasive means of quantitatively assessing changes in BBB permeability and GS function in individuals with vascular cognitive impairment.

### Assessment of BBB permeability changes

**Figure 2:**
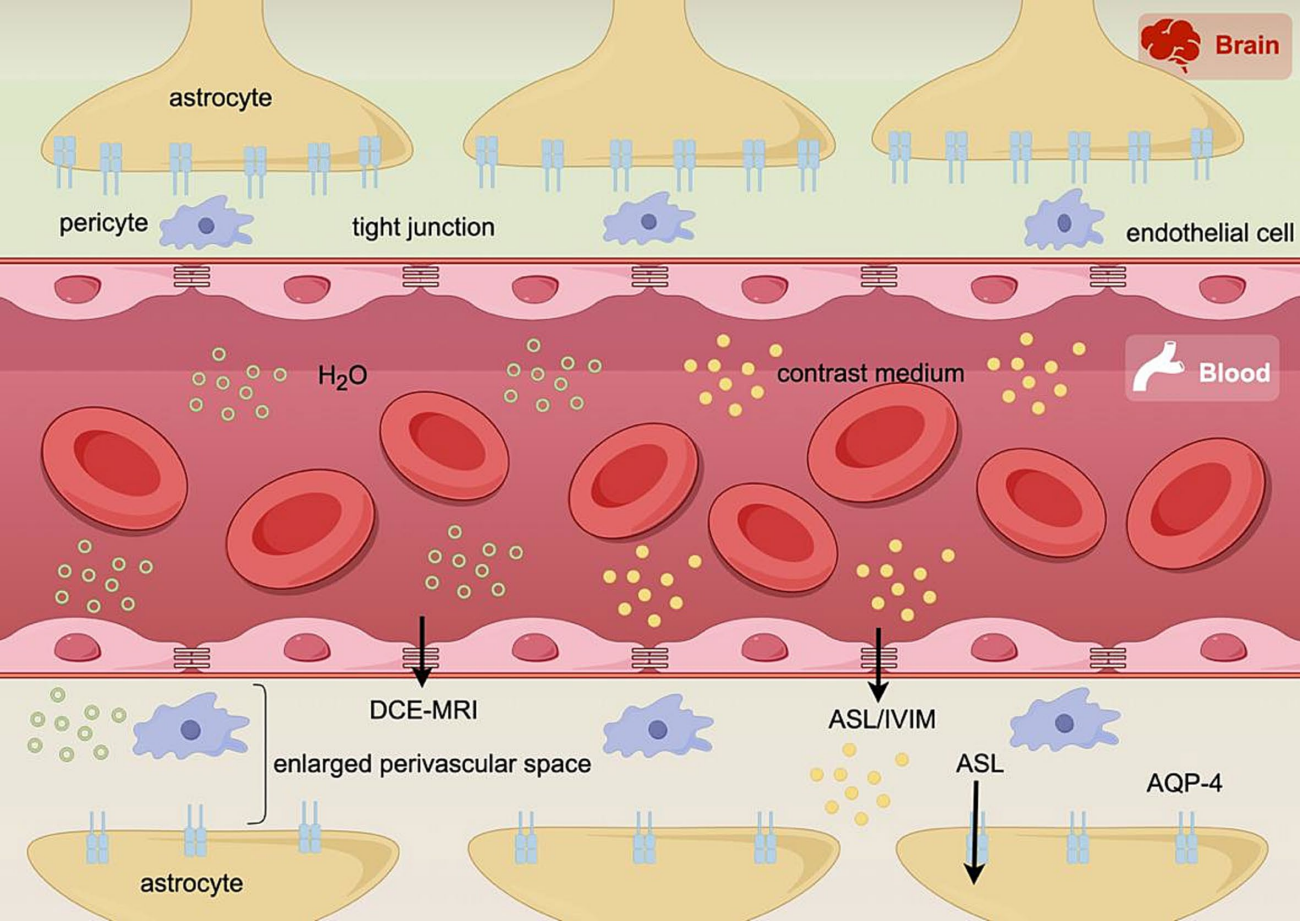
Schematic representation of the blood–brain barrier (BBB) and perivascular space (PVS): when the BBB permeability increases and the PVS widens, the rate of passive transport of contrast agent and water molecules from the intravascular to the extravascular space increases; at the same time, decreased aquaporin-4 (AQP-4) in astrocyte foot segments, which decreases the active transport of water molecules within the PVS and also makes the PVS wider and the GS dysfunction. These pathological changes can be characterized by the quantitative parameters of dynamic contrast-enhanced MRI (DCE-MRI) requiring exogenous contrast agent and arterial spin-labeled MRI (ASL) and intravoxel incoherent motion MRI (IVIM) without exogenous contrast agent, respectively. Images were produced using FigDraw software (www.figdraw.com) (DOI: AYSRP3949a).

In the contemporary era, MRI brain perfusion weighted imaging (PWI) sequences have garnered significant attention as a focal point of research for evaluating alterations in BBB permeability. PWI can be categorized into two distinct types, one of which necessitates the use of exogenous contrast agent and encompasses dynamic susceptibility contrast MRI (DSC-MRI) and dynamic contrast-enhanced MRI (DCE-MRI). The other category of sequences for PWI is arterial spin-labeling (ASL) based MRI without the usage of exogenous contrast agents, which utilizes hydrogen protons in labeled arterial blood as an endogenous and freely diffusible tracer (Williams et al., 1992; Johnston et al., 2015). Intravoxel incoherent motion imaging (IVIM) can also provide perfusion information without the need for exogenous contrast agents (Federau et al., 2012) (As shown in Figure 2).

DSC-MRI employs the BBB model to acquire semi-quantitative parameters such as cerebral blood flow (CBF), cerebral blood volume (CBV), mean transit time (MTT), and time to peak (TTP) (Kouwenberg et al., 2022; Boxerman et al., 2020); while DCE-MRI is primarily utilized for the assessment of vascular permeability, with key parameters including semi-quantitative parameters such as area under the curve (AUC), TTP, maximal signal intensity, maximal slope, and rate of contouring, etc., and quantitative parameters such as the volume transfer constant (K^trans^), the rate constant (K_ep_), and the fractional interstitial volume (V_e_), etc. (Thrippleton et al., 2019). DCE-MRI is commonly utilized in clinical research as the preferred modality for evaluating changes in BBB permeability (Elschot et al., 2021). Several previous studies (Wang et al., 2023; Kerkhofs et al., 2021a) have confirmed that BBB disruption may be associated with the development of VCI and may precede the appearance of white matter lesions by quantitative parameters of DCE-MRI. A longitudinal study of Kerkhofs et al. (2021b) found that CSVD patients with higher baseline BBB permeability had more severe cognitive decline, further confirming the critical role of BBB disruption in the development of cognitive impairment.

ASL-labeled water molecules cross the BBB and passively diffuse into the brain tissue through tightly connected endothelial cell layers and active transport proteins (e.g., AQP-4 channels on astrocytes). Three modules were commonly used for the water labelling, namely, pulsed ASL (pASL), continuous ASL (CASL) and pseudo-continuous ASL (pCASL) (Fan et al., 2016; Wintermark et al., 2005). ASL-related techniques have the advantages of being non-invasive, contrast-free, and reproducible. ASL quantifies BBB permeability by water molecule transfer rate (Kw) and cerebral perfusion by CBF. Sun et al. (2016) observed a diffuse decrease in CBF in the brain of the SVCI group compared with HC group, which is similar to the findings of Schuff et al. (2009). A longitudinal study of van Dinther et al. (2024) found that lower baseline CBF was associated with cognitive decline in patients with VCI, an association that was most pronounced in attention/psychomotor speed, and that lower temporal and frontal CBF at baseline was associated with more severe memory loss. Neumann et al. (2022) also found that CBF decreases and arterial transit time (ATT) increases as cognitive severity increases in patients with CSVD. However, whether the correlation between CBF and cognitive level in patients with VCI, derived from longitudinal follow-up studies and clinical prediction modeling, is sufficient to indicate a causal relationship remains to be further investigated. In addition, the interference of factors such as cardiovascular disease that may accompany the increase in age makes the choice of post-labeling delay time (PLD) to have some impact on the results of the study. Thus, the commonly used PLD time of 1,800 ms may underestimate CBF in patients with cerebrovascular disease, and the ability to assess the correlation between CBF and the degree of cognitive impairment may be less accurate. Therefore, CBF alterations in VCI patients can be assessed using ASL sequences with multiple PLD times. The ASL sequence uses water molecules as the endogenous contrast agent, which has a much lower molecular weight than the contrast agent of the DCE-MRI sequence and can diffuse more readily into the BBB (Shao et al., 2020). Therefore, theoretically, altered water transport across the BBB should be detected earlier than contrast leakage. Finally, IVIM sequences can obtain perfusion coefficient (D*) value and perfusion fraction (f) value to reflect the microcirculatory perfusion and vascular permeability within the capillaries under low b-value conditions, which can also be used to assess the alteration of BBB permeability in VCI patients (Le Bihan et al., 1988). It can be seen that both types of PWI imaging sequences above can obtain information reflecting perfusion and BBB permeability, which provides an effective tool for studying cerebral underperfusion, cerebrovascular reactivity and BBB permeability in patients with VCI.

### Evaluation of alterations in the activity of the glymphatic system

With the development of MRI techniques, the assessment of changes in GS activity can be objectively presented by quantitative MRI techniques, including MRI techniques with or without requirement of tracer injection. Intrathecal tracer injection is generally used to assess GS activity through animal models, which can reflect the GS clearance function over a long period of time, and is not affected by the BBB and blood circulation, but this method is invasive and prone to tracer deposition in the brain (Provenzano et al., 2019); intraventricular tracer injection is susceptible to the BBB and blood circulation, but is safer than intrathecal tracer injection and more commonly used in human beings (Mortensen et al., 2019; Kaur et al., 2020). MRI methods requiring tracer injection are based on the distribution of tracer in tissues and tissue characteristics to establish a pharmacokinetic model to simulate the process of MRI signal changes to achieve the assessment of GS activity, however, they are susceptible to the influence of magnetic field strength, magnetic field uniformity, and scanning parameters. Tracer-less methods, such as the dispersion correlation sequences, can be used for non-invasive assessment of GS activity due to their ability to track the movement of water molecules (Voorter et al., 2024). Besides, IVIM sequences can indirectly reflect fluid mobility within the PVS by changes in D* and f values under low b-value conditions; unfortunately, this method is susceptible to the influence of intercellular ISF (van der Thiel et al., 2021; Wong et al., 2020). Diffusion tensor imaging analysis along the perivascular space (DTI-ALPS) assesses the movement of water molecules in the direction of the PVS as measured by the diffusivity of water molecules measured by DTI sequences (Taoka et al., 2017). The DTI-ALPS analytical method can be used to express the ability of water diffusion along the direction of the PVS by means of the ALPS index, reflecting the GS activity of an individual patient: smaller ALPS index reflect, to some extent, GS impairment. However, this method is only applicable to areas adjacent to the lateral ventricles. Recently, several studies (Ke et al., 2022; Liang et al., 2023; Tian et al., 2023) have found that ALPS index were significantly lower and significantly associated with overall cognitive scores in patients with CSVD by the DTI-ALPS method; furthermore, a DTI-ALPS study (Song et al., 2022) in patients with SVCI showed that structural network efficiency mediated the link between impaired GS function and cognitive dysfunction in patients; Xu et al. (2022) analysis found that patients with cerebral amyloid angiopathy had reduced ALPS index and were associated with lower cognitive scores compared with HC group. In conclusion, GS dysfunction may be a potential pathologic mechanism for vascular cognitive dysfunction, and the ALPS index is expected to be an emerging MRI marker for it. However, the DTI-ALPS method needs to be aligned with magnetically sensitive weighted imaging (SWI). Zhang et al. (2021) performed a modification to obtain a modified ALPS (mALPS) index to reflect GS activity without SWI alignment. These DTI-based analysis methods have the advantages of being noninvasive, tracer-free, and requiring only one scan, but they can only reflect GS transient activity. There are also studies to quantify GS activity by ASL sequence (Ohene et al., 2019) and phase contrast method (Ichikawa et al., 2018). It is believed that with the continuous development of MRI imaging technology, more reliable methods to quantify GS activity can be provided.

MRI-visible perivascular spaces (PVSs) (Bouvy et al., 2014), free water (FW) fraction (Kamagata et al., 2022), and choroid plexus (ChP) volume (Zhang et al., 2021) had also been used to assess the GS function. The PVS is a fluid-filled cavity surrounding small blood vessels in the brain that aids in the exchange of ISF and CSF for waste removal (Brown et al., 2018). Enlarged perivascular spaces (EPVS) are characterized by round or oval smooth-edged structures larger than 2 mm in the perforating artery supply area, exhibiting equal signal intensity to CSF on MRI (Wardlaw et al., 2020). The early detection of PVS morphology is contingent upon the spatial resolution of imaging scans, with ultrahigh-field MRI offering the potential for higher resolution and the ability to detect smaller PVS at an early stage (Barisano et al., 2021). In the clinical assessment of the number of PVS, visual rating scales are usually chosen for this purpose (Rowley, 2013; Laveskog et al., 2018), and the most widely used one is the STRIVE criterion developed by Wardlaw et al. (2013): the quantification of PVS is conducted in the basal ganglia and the centrum semiovale, with classification into mild, moderate, and severe categories based on PVS count. However, the STRIVE criterion is subject to several limitations: (1) challenges in distinguishing between PVS and other cerebrovascular disease markers like lacunar infarcts or white matter lesions; (2) the time-consuming and subjective nature of manual counting, which increases the risk of detection bias; (3) the evaluation of PVS necessitates the utilization of high-resolution MRI images to ensure accuracy, limiting the feasibility of obtaining additional clinically relevant metrics, such as PVS volume; (4) the visual scale method is not suitable for longitudinal studies. In light of the aforementioned challenges, an increasing number of scholarly investigations (Dubost et al., 2019; Moses et al., 2023; Barisano et al., 2022) have been dedicated to the automated quantification of PVS, utilizing techniques such as intensity-based thresholding (Schwartz et al., 2019; Boespflug et al., 2018; Niazi et al., 2018), vascular filtering-based (Ballerini et al., 2018; Sepehrband et al., 2019), and machine learning (Dubost et al., 2019; Lian et al., 2018; Boutinaud et al., 2021). These methods are characterized by their speed, convenience, objectivity, and reproducibility. EPVS, a neuroimaging biomarker observed in individuals with CSVD, is closely linked to cognitive dysfunction. Zdanovskis et al. (2022) found that individuals in the SVCI group exhibited greater expansion of PVS in the centrum semiovale, basal ganglia, and midbrain regions compared to the HC group using visual rating scales. Future research utilizing automated quantification methods is anticipated to further investigate and assess early alterations in PVS number and volume in patients with VCI. ChP constitutes a critical component of the BBB (Damkier et al., 2013) with primary function is the production of cerebrospinal fluid; however, it also plays a pivotal role in mediating the brain’s clearance pathway, thereby contributing to the maintenance of cerebral homeostasis (Rees et al., 2016) and the functioning of the GS (Jessen et al., 2015). Meanwhile, recently, the FW diffusion MRI model has gained particular attention in CSVD (Duering et al., 2018; Li et al., 2024) as well as other brain diseases (Kamagata et al., 2022; Nägele et al., 2024). This model can be divided into FW compartment and tissue compartment, the FW compartment represents water molecules that are not restricted or directed. It is modeled by a tensor that is isotropic and has a fixed diffusion coefficient of water at 37°C. The tissue compartment represents all remaining water molecules, i.e., water molecules within or in close proximity to cellular structures. This includes intracellular water as well as extracellular water affected by physical barriers, such as axon membranes and myelin. Hence, the tissue compartment reflects white matte fiber organization (Duering et al., 2018).

## Analysis of altered neurovascular coupling (NVC) function

The neurovascular unit (NVU), which consists of neurons, astrocytes, vascular smooth muscle cells (SMCs), endothelial cells (ECs), and pericytes (Abbott et al., 2006; Tam and Watts, 2010), plays a crucial role in the coupling of vascular and neuronal activity and is of increasing interest in the pathophysiology of VCI. Disruption of any neurovascular coupling (NVC) function component affects CBF regulation and neuronal function (Kisler et al., 2017). Thus, intact NVC function is critical and even minor damage can affect brain function and lead to cognitive decline (Toth et al., 2017). Recently, the analysis of NVC based on ASL and resting-state functional MRI (rs-fMRI) sequences has provided new perspectives for obtaining the functional status of NVC. Ruan et al. (2023) analyzed the associations between white matter lesion (WML) burden, cognitive function and NVC coefficients using mediation methods among the SVCI, PVCI, and HC groups, and found that compared with HC, NVC was significantly lower in the SVCI and PSCI groups at both whole-brain and brain-region levels, and NVC coefficients were also found to be lower in higher-order brain systems responsible for cognitive control and emotion regulation. Mediation analysis suggest that NVC mediates the relationship between WML burden and cognitive impairment. Liu et al. (2021) combined regional homogeneity (ReHo) values obtained by rs-fMRI post-processing with CBF from ASL sequence, using global ReHo-CBF correlation coefficients and ReHo/CBF ratios to detect an intrinsic link between neuronal activity and vascular responses in patients with SVCI, and found that patients in the SVCI group showed significantly lower overall ReHo-CBF coupling, as well as a markedly abnormal ReHo/CBF ratio mainly located in cognitively relevant brain regions and the ReHo/CBF ratio in the left precentral gyrus was positively correlated with the MMSE score. These findings suggest that patients with VCI exhibit abnormal neurovascular coupling early in the disease and during disease progression, which may be related to disease severity and cognitive impairment.

## Assessment of brain connectivity

### Connectivity analysis based on resting-state functional MRI of the brain

Rs-fMRI sequence is used to detect spontaneous brain activity in the resting state to explore brain function, by detecting spontaneous neuronal activity in the baseline state of the subject’s brain and determining the network connectivity of the relevant brain regions (Raimondo et al., 2021). Currently, the application of rs-fMRI sequence in evaluating brain connectivity in VCI patients is mainly divided into two categories: The first category is the indicators reflecting brain connectivity in the resting state: functional connectivity (FC), functional network connectivity (FNC) and graph theory analysis; and the second category is the connectivity analysis methods reflecting the dynamic change process of the brain: dynamic functional connectivity (dFC), dynamic functional network connectivity (dFNC), graph theory analysis, gradient analysis of brain networks, Granger causality analysis (GCA), multilayered brain networks, and other analysis methods.

### Static brain functional connectivity and functional network connectivity analysis

Resting-state brain networks are generally analyzed by FC, FNC and graph theory analysis, in which FC and FNC are obtained by calculating the degree of correlation between two time series, positive correlation represents the existence of positive connections and vice versa, whereas the graph theory approach is based on FC, FNC for topological analysis.

The study of FC can be categorized into the voxel level and the ROI level. All of these FC analysis methods are based on linear models, while some studies have found that BOLD signals have nonlinear characteristics, therefore, FC analysis methods based on nonlinear models to obtain parameters such as maximum information coefficient (MIC), extended maximum information coefficient (EMIC), and so on, may be more suitable for the use of rs-fMRI to capture the complex interactions between brain regions. Chen et al. (2019) found that patients with SVCI had reduced FC between default mode network (DMN)-related brain regions and reduced negative connectivity with other network brain regions by the seed-based correlation analysis (SCA) method, at the same time, patients with SVCI were found to have impaired memory function associated with increased FC between DMN-related brain regions in the medial frontal lobe, whereas the patients’ impaired information processing was associated with reduced FC between DMN-related brain regions in the thalamus. Recently, other studies (Zuo et al., 2019; Sun et al., 2011) have found different trends of FC changes in different brain regions in patients with VCI, possibly due to subcortical ischemia disrupting directly and indirectly connected fiber bundles, which triggers weakened connectivity in some brain regions, whereas enhanced connectivity in some brain regions may be caused by compensatory compensation for cognitive impairment and neuroplasticity mechanisms in functional brain regions.

FNC analysis employs a strategy that combines model-driven and data-driven approaches (Jafri et al., 2008). First, grouped independent component analysis (ICA) is performed on rs-fMRI data from multiple subjects to derive subject-specific functional networks called resting-state networks (RSNs), commonly known as DMN, central executive network (CEN), salience network (SN), frontoparietal network (FPN), sensorimotor network (SMN), visual network (VN) and auditory network (AN) etc. (Mueller et al., 2014). Finally, the connectivity between any RSNs can then be obtained by calculating the connectivity metric, resulting in a connectivity matrix that includes the strength of connectivity between all networks. Wei et al. (2024) found different trends of changes in different RSNs interconnection models were found, along with different correlations between different RSNs interconnection models and MoCA scores, suggesting that there is a correlation between FNC changes and cognitive deficits in SVCI patients. Chen H. et al. (2023) also found increased FNC change between the VN and cerebellar domains in patients with SVCI, however, there was a reduction in FNC change between the cognitive control network and the cerebellar domains and a significant reduction in FNC within the sensorimotor domain. These studies also illustrate that the FNC approach can provide a more detailed delineation of brain regions reflecting the temporal connectivity between different brain regions by using a higher model order. In contrast to ROI-based approaches, FNC analysis employs a data-driven approach to generate functionally co-activated brain regions, whereas brain regions in ROI-based approaches are typically determined through *a priori* knowledge (Du et al., 2018).

In functional brain network analysis, relevant attributes such as local efficiency (Eloc), global efficiency (Eg), clustering coefficient (Cp), standardized clustering coefficients (γ), small-world attributes, characteristic path length (Lp), etc., can be obtained from graph theory analysis to evaluate the topological properties of brain networks (Farahani et al., 2019). Graph theory analysis helps to describe and quantify the topology of the brain, and can identify key nodes that are important and functional in the brain network. Several studies (Sang et al., 2018; Wang et al., 2019) have found that with the increase of cognitive impairment in VCI patients, the pattern of brain functional connectivity network is disrupted and the graph theory related properties are changed by rs-fMRI graph theory analysis method, which also indicates that the graph theory based brain functional network analysis method can provide a potential imaging biomarker for the early detection of cognitive impairments in VCI patients, however, most of the studies are descriptive and focus on pairwise interactions of binary compositional graphs, as the technology develops, the generative model, dynamic network, and algebraic topology will be the several promising directions for the future development.

### Dynamic functional connectivity and functional network connectivity analysis

In recent years, more scholars believe that the brain’s information transfer is time-dynamic and condition-dependent (Chang and Glover, 2010), so purely relying on static analysis methods can not accurately describe the dynamic changes in the brain, and dynamic analysis methods can provide new perspectives on the dynamic functional activities of the brain: including dFC, dFNC, and the dynamic graph theory analysis, in addition to the analysis of the gradient analysis of the brain network, GCA, multi-layer brain network and other analytical methods.

dFC and dFNC are based on FC, ICA (Kiviniemi et al., 2011) and independent vector analysis (IVA) (Calhoun et al., 2014), using sliding time window (Madhyastha and Grabowski, 2014; Maes et al., 2022), joint time-frequency analysis (Yaesoubi et al., 2015) and other methods to transform the RSN into dynamic brain network, and then through K-means clustering and Gaussian mixture model clustering analysis to obtain multiple states, and finally to calculate the fractional occupancy of each state, the mean dwell time, the lifetime switching rate, and the transition probability matrix to realize the dynamic analysis of brain function. Currently, dFC analysis methods are mainly divided into two paths, one is to capture synchronous changes in paired regions, such as sliding window analysis and time-frequency analysis; the other is to identify synchronous change patterns at the multivariate level, such as co-activation (CAP), hidden Markov model (HMM), and multilayer brain network. Recently, fractional occupancy and mean lifetime of brain states in patients with SVCI have been found to be related to cognitive performance, as evidenced by higher fractional occupancy and longer mean lifetime in weakly-connected states and greater difficulty in switching from weakly-connected to more strongly-connected states (Sang et al., 2023). Xu et al. (2021) used dFC analysis method to categorize the brain states into seven different states, and observed that cognitive deficits in patients of state 3 with SVCI predominantly reduced the mean dwell time, with a strong overall positive correlation. Yin et al. (2022) analyzed the relationship between the temporal characteristics of dFC and cognitive impairment in patients with SVCI, and they classified the dFC status into sparse connectivity, strong connectivity, and intermediate patterns by K-means clustering analysis, and found that SVCI patients showed a higher fractional occupancy and longer mean dwell time in sparse connectivity, and a lower fractional occupancy and shorter dwell time in strong connectivity, which was similar to the finding of Chen F. et al. (2023). Meanwhile, this finding suggests that altered temporal properties of dFC in patients with SVCI may be a sensitive neuroimaging biomarker for early disease recognition. In addition to the above construction of dFC by the sliding window method, Schlemm et al. (2022) constructed dFC based on the co-activation pattern method, obtained DMN activation state, DMN inhibition state, VN activation state, VN inhibition state, and FPN state of the patients with SVCI by clustering analysis, and found that cognitive impairments were associated with the convergence of the mean dwell time of each state and the decrease of gradient between each pattern. Consistent sets of brain states and spatial alterations in functional connectivity that correlate with different states of consciousness or awareness can be identified by dFC, which provides additional options for analyzing brain connectivity in patients with VCI.

By integrating the aforementioned methods for analyzing dFC and dFNC, it has been observed that while the introduction of windows facilitates a dynamic assessment of brain connectivity, the selection of window length significantly influences the outcomes. Specifically, shorter window lengths tend to stabilize the relationship between the time series of two nodes within the window, thereby potentially increasing the calculated correlation. This phenomenon explains why dFC often yields more significant results, as static FC is analogous to using a window length equivalent to the entire scanning duration. However, it is important to note that correlations derived from excessively short window lengths may not accurately reflect genuine brain activity (Leonardi and Van De Ville, 2015). Furthermore, the optimal window length for assessing dFC between various nodes may vary. Zhang et al. (2018) proposed that a shorter window length is appropriate for analyzing intra-network connectivity, while a longer window length is preferable for examining inter-network connectivity. This distinction may arise from the influence of window length on the frequency of functional connectivity that can be detected. Specifically, a shorter window length is more conducive to observing connectivity associated with high-frequency signals, whereas a longer window length is more suitable for detecting connectivity related to low-frequency signals. Consequently, it is crucial to develop dynamic brain function analysis methods that mitigate the influence of window length. The CAP model addresses this by assessing the activation patterns of the entire brain at each sampling point, thereby describing the brain’s dynamic function in terms of instantaneous network states, which reflect the brain’s dynamic function at the smallest time scale (Liu and Duyn, 2013; Liu et al., 2018); the HMM model effectively solves the problem of the time window configuration by characterizing the brain activity as a sequence of different states deduced from the resting data problem (Zhang G. et al., 2020; Vidaurre et al., 2017); and the multilayer brain network analysis method can estimate a neighbourhood matrix for each time window, interconnecting all windows into a multilayer network, with each time window represented as a layer. This method constructs a hyper-adjacency matrix that captures both intra- and inter-layer connections, thereby offering a comprehensive perspective on dynamic interactions (Pedersen et al., 2018).

### Properties analysis of DTI brain networks based on graph theory

Graph theory-based DTI brain network property analysis is based on brain partition mapping for deterministic or probabilistic fiber tracking between multiple brain regions two by two, using specific brain regions as nodes and structural connectivity indicators (fiber bundle bar count, probability, average FA value, etc.) between nodes as edges, which can construct structural brain networks and calculate small-world properties, Rich-club coefficients, degree centrality, etc., and utilize the graph theory to calculate the corresponding network properties to analyze brain structural connectivity (Rubinov and Sporns, 2010; Sporns, 2018). Kim et al. (2015) assessed the cognitive status of SVCI patients based on DTI graph theory analysis, and observed that an increase in white matter high-density volume or number of lumens was significantly associated with a decrease in network integration and an increase in network separation, and that changes in these network attributes were associated with poor performance of attention, language, visuospatial, memory, and frontal executive function performance was associated with poor performance. Tuladhar et al. (2017) also observed reduced structural network density, network strength, and efficiency in SVCI patients, and that the reduced connectivity was mainly manifested in the connectivity between rich-club nodes, with lower rich-club connectivity associated with lower psychomotor speed and executive function scores. Graph theory-based analysis of the structural network properties of the DTI brain can be used as a complement to the graph theory analysis of FC, and has some clinical applications in the assessment of patients with VCI.

## Assessment of metabolic changes

Traditional proton-based magnetic resonance spectroscopy (^1^H-MRS) is capable of identifying metabolites such as choline (Cho), N-acetylaspartate (NAA), and creatine (Cr) within brain tissues *in vivo*, and has been extensively utilized for evaluating metabolic changes in the brains of individuals with VCI ((Barry Erhardt et al., 2019; Chen et al., 2016; Ross et al., 2005; Lin et al., 2021). Nonetheless, the complexity of the background, overlapping spectra, and the presence of pressurized water/lipids inherent to the ^1^H-MRS technique restrict its utility as a molecular imaging tool, hindering the ability to capture dynamic molecular metabolic processes. The emergence of multinuclear MRI, facilitated by advancements in MRS technology, enables the acquisition of diverse metabolite information within the human body, thereby augmenting the precision of detecting both physiological and pathological processes. This technology maintains spatial resolution while offering the unique capability of noninvasive, in vivo quantitative analysis of dynamic molecular metabolic processes, including the accurate quantification of energy metabolic pathways such as anaerobic glycolysis and the tricarboxylic acid cycle, and at the same time, can detect tissue osmotic pressure, neurotransmitters and mitochondrial function (Sun et al., 2023). Multinuclear MRI offers a distinct advantage in visualizing real-time dynamic metabolic processes across various pathological conditions. This technology is anticipated to offer novel opportunities for early disease detection, treatment efficacy evaluation, therapeutic decision-making, and drug development. Furthermore, it has the potential to facilitate the exploration of novel pathogenic mechanisms and is poised to become a valuable tool for advancing molecular biology, biochemistry, and metabolism research in human subjects.

Amide proton transfer weighted imaging (APTw) is an MRI technique utilizing chemical exchange saturation transfer (CEST) to assess protein properties at the cellular molecular level within tissues. The APTw sequence offers non-invasive, safe, and quantitative analysis, with changes in APT signal primarily influenced by the exchange rate between amide protons and water molecules. This exchange rate is closely linked to the concentration of proteins and peptides in the body, as well as the pH value (Milot, 2022; Zhou and Van, 2006).; APTw imaging has been extensively utilized in various areas of neuroscience research, including brain development (Zhang Z. et al., 2020; Zhang et al., 2016), cerebral vascular diseases (Sun et al., 2011; Yu et al., 2019), brain tumors (Joo et al., 2019; Guo et al., 2022), and neurodegenerative diseases (Chen X. et al., 2023; Guo et al., 2021), etc. Additionally, investigations have been conducted to determine if APT values could serve as a potential indicator of VCI. Mu et al. (2023) has demonstrated statistically significant differences in APT values of specific brain regions, such as the frontal white matter, hippocampus, amygdala, and thalamus, suggesting their potential utility as markers for VCI.

Sodium-23 MRS (23Na-MRS) is the sole noninvasive method capable of providing precise spatial quantification of sodium concentration in living tissue through imaging technology. This technique provides direct biochemical insights into cellular integrity and tissue viability, with minimal observable macroscopic alterations. Consequently, it can be utilized to monitor temporal changes in tissue viability during treatment and potentially serve as a biomarker for early preventative diagnosis in clinical settings (Madelin et al., 2014; Chen et al., 2022). ^23^Na-MRS has been used in neurological diseases such as AD, Huntington’s disease (HD) and multiple sclerosis (MS) (Reetz et al., 2012; Mellon et al., 2009). These investigations highlight the promise of ^23^Na-MRS for the timely detection and tracking of disease advancement and treatment efficacy in various neurological disorders, as indicated by the observation of heightened tissue sodium concentration (TSC) in the brain. Phosphorus-31 MRS (31P-MRS) enables noninvasive monitoring of cell membrane proliferation, mitochondrial processes involved in energy metabolism (Rzanny et al., 2003), tissue acid–base balance, tissue magnesium ion concentration, and specific nucleotides (Larkin et al., 2021). Zuo et al. (2015) identified variances in phosphocreatine (PCr) and phosphoric acid (Pi) levels through ^31^P-MRS assessments between individuals diagnosed with depression and a HC group, while Dudley et al. (2015) noted a notable decrease in PCr and adenosine triphosphate (ATP) levels in the gray matter of individuals with bipolar disorder (BD) using ^31^P-MRS. These findings underscore the utility of ^31^P-MRS in measuring mitochondrial metabolic irregularities. Meanwhile, ^31^P-MRS has shown promise as a valuable tool for assessing treatment response by serving as a biomarker for phospholipid metabolism. Specifically, the phospholipid monolipid (PME)/phospholipid diolipid (PDE) ratio in individuals with AD demonstrated an increase following a 4-week course of Souvenaid treatment (Rijpma et al., 2017). On the other hand, ^31^P-MRS has been utilized in research examining pH fluctuations in individuals post-traumatic brain injury (TBI) (Stovell et al., 2020). Hyperpolarized-^13^C-MRS enables the *in vivo*, non-invasive quantitative evaluation of key biochemical processes such as the tricarboxylic acid cycle and glucose metabolism in the human body (Mandal et al., 2022). Research utilizing hyperpolarized-^13^C-MRS has shown increased pyruvate-to-lactate conversion in longitudinal studies of MS mouse models (Guglielmetti et al., 2017b) and in acute and subacute phases of TBI mouse models (Guglielmetti et al., 2017a; DeVience et al., 2021). These findings offer valuable insights for assessing the effectiveness of anti-inflammatory treatments and managing patient care. The investigation into the utility of multinuclear MRI for evaluating changes in localized metabolic substances in the brains of patients with VCI is currently in its early stages. It is anticipated that advancements in magnetic resonance hardware systems, fast sequence methods, and accelerated algorithms such as compressed sensing and deep learning will contribute to the maturation of multi-nuclear MRI/MRS as a valuable tool for assessing metabolic alterations in the entire brain or specific regions of interest in patients with VCI, in the meantime, further research is needed to investigate the potential benefits of incorporating joint analysis with other functional MRI sequences and brain network analysis.

## Summary and outlook

VCI, as a group of diseases mainly caused by cerebrovascular diseases, undergoes a progression from mVCI to VaD throughout the course of the disease, with structural, functional, metabolic, and cerebral connectivity changes occurring in different brain regions throughout the process, and with the development of neuroimaging, the comprehensive assessment and understanding of these changes has been greatly facilitated. However, we have found that the variation in results across studies may be related to a variety of factors such as patient selection and sample size, MRI field strength, scanning protocols or reproducibility on different scanning devices, and feasibility of implementation, in addition to the fact that patients may be at different stages of the disease, and a standardised protocol is therefore needed to increase the reproducibility of relevant studies, as in the case of the HARNESS protocol which standardises the effects of vascular factors on dementia and neurodegeneration effects on brain imaging protocols, contributing to increased study reproducibility. With the development of ultra-high field strength MRI equipment, which relies on its high resolution, good contrast between tissues and better signal-to-noise ratio to facilitate the detection of subtle lesions as well as finer blood vessels, it is becoming a new tool for CSVD studies. However, these new techniques still need to be validated to define their operational specifications before they can be widely disseminated. In addition, the application and development of multimodal MRI, neural networks, machine learning, and deep learning in image processing can further increase diagnostic accuracy, enabling the extraction of more imaging information from data that would otherwise not be easily accessible to provide additional support for diagnosis.

In addition, most of the neuroimaging studies on VCI are cross-sectional studies, and there is a lack of longitudinal studies to validate the results, so the longitudinal development and changes in the structure, function, metabolism, and connectivity of patients with VCI should be explored more deeply in future studies. Finally, with the exploratory development of transcriptional connectome association studies, it provides an important opportunity to bridge the gap between microscopic transcriptome features and macroscopic brain networks. However, the underlying molecular mechanisms of altered brain function associated with VCI patients remain unclear and await further exploration in future studies.
